# Supplementary material for: Duodenal Dysbiosis and Relation to the Efficacy of Proton Pump Inhibitors in Functional Dyspepsia
Source: Int J Mol Sci. 2021 Dec 19;22(24):13609. doi: 10.3390/ijms222413609 (PMC8708077; doi:10.3390/ijms222413609)
Supplement: Supplementary file 1 [file ijms-22-13609-s001.zip › ijms-1507068-supplementary.pdf]

SUPPLEMENTARY RESULTS

Study cohort and sample overview

An overview of the included subjects and collected samples with high-quality sequences is given in **Table S1**. Besides 2 drop-outs (1 FD-starter and 1 FD-stopper with no follow-up visits), variability was assessed in 19 FD-starters and 25 controls after amendment of the protocol. Due to technical difficulties during endoscopy, brushes were missing for 2 baseline and 3 follow-up visits. After sequencing and quality control, 1 additional brush and biopsy sample were lost for each visit. Based on the high number of reads with brushes (**Figure S1A**), a subanalysis was done with minimal 10,000 reads, similar to fecal microbiota analyses.[12,14] While only 1 biopsy sample was discarded using a cut-off of 1,000 reads, only 3 brush but 62 biopsy samples were discarded using a cut-off of 10,000 reads (**Table S1**). From the 185 brushes and 130 biopsies with >10,000 reads, a total of 696 and 564 annotated genera were obtained after sub-setting and CLR-transformation, respectively.

Median (IQR) read number was 4,496 (1,501-11,204) for negative controls (**Figure S1A**) with no sequenced reads for no-primer controls. Variability between plates was assessed using the Bray-Curtis dissimilarity of rarefied (1,000 reads) positive and universal controls, which was <.3 (**Figure S1B**). Microbial load of brushes and biopsies was studied in mixed models with no significant main effects of group and treatment or interaction effects.

Duodenal microbiome is altered in FD patients with PPI-effects

Significant associations of sampling location (R^2= 15.74%, P_{adj}= .007), subject (R^2= 2.39%, P_{adj}= .07) and group (R^2= .35, P_{adj}< .1) but not PPI or demographics with duodenal community variation were found using univariate dbRDA for all samples (n= 380). Although only the contribution of location remained significant in a multivariate model (R^2= 22.14%, P_{adj}= .002), this could be driven by the persisting presence of potential contaminants in biopsy samples even after decontam (see below). A significant contribution of location (R^2= 17.84%, P_{adj}= .007) but not subject was also found using a cut-off of 10,000 reads (n= 315), and possibly driven by the presence of contaminants in biopsy samples. Principle component analyses with effect sizes (PERMANOVA) of location (all samples), PPI and group (also for brush and biopsy samples separately) are shown in **Figure S2**.

Specific effects on genera and diversity after short-term PPI

Main and interaction effects of mixed model analyses for mucus-associated genera of interest and α-diversity are shown in **Table S2.** Using a cut-off of 10,000 instead of 1,000 reads, the lower abundance of mucus-associated *Neisseria* (FDR< .001), *Porphyromonas* (FDR< .01), *Selenomonas* (FDR= .06) and *Haemophilus* (FDR= .06) was confirmed in FD-starters vs. controls with decreased *Prevotella* (FDR= .09) after PPI. Although changes in mucus-associated richness were lost, the decrease in Shannon and Simpson's index remained significant in controls (all *P*< .01) and FD-starters (all *P*= .01) after PPI. In the absence of differentially abundant epithelium-associated genera after correction for multiple testing, no mixed model analyses were done. For mucosal α-diversity, a significant group effect was found for richness (F= 3.42, *P*= .04), driven by changes in FD-stoppers (see below). No other main or interaction effects were found.

Regarding spatial variation, all 45 genera (taxonomically assigned and prevalence >20%) were differentially abundant (all FDR< .1) but with a higher abundance of potential contaminants in the duodenal biopsy samples (**Figure S3**).[16] In addition, similar richness but lower diversity was found for brush vs. biopsy samples in both groups and according to PPI (**Table S3**). In contrast, genera abundance was similar between baseline and variability visits off-PPI (FDR≥ .1) and with no within- or between-group differences in α-diversity metrics for duodenal brush or biopsy samples (**Table S4**). Results were similar when using a cut-off of 10,000 reads, with significant spatial variation of all genera (all FDR< .1) and lower Shannon and Simpson's index in brush vs. biopsy samples of controls (all *P*< .0001) and FD-starters (*P*= .001 and *P*= .0001, respectively) and no significant temporal variation (all *P*> .05).

Persisting microbiota alterations after withdrawal of long-term PPI

A lower abundance of epithelium-associated *Mesorhizobium* (FDR< .01), *Sediminibacterium* (FDR= .06) and *Dyella* (FDR< .1) was found in FD-stoppers vs. controls, although the first 2 are potential contaminants.[16] *Dyella* decreased after PPI-withdrawal (β= -2.09 ± .93, *P*= .03) and was lower in FD-stoppers vs. controls off-PPI (β= -3.56 ± 1.08, *P*=.002) with a significant difference between the changes in FD-stoppers vs. controls (β= -2.98 ± 1.17, P_{adj}= .04) or interaction effect (**Figure S4A**). Using a minimum number of 10,000 reads, the decrease in mucus-associated *Rothia* (FDR= .02) and *Stomatobaculum* (FDR= .08) but not epithelium-associated *Dyella* was confirmed after PPI-withdrawal in FD-stoppers.

Changes in mucus- and epithelium-associated α-diversity metrics after withdrawal of long-term PPI in FD-stoppers and the comparison with controls and FD-starters are shown in **Table S5**. The group effect for epithelium-associated richness was explained by lower values in FD-stoppers vs. FD-starters (β= -.17 ± .08, *P*= .03) and controls (β= -.2 ± .07, *P*< .01) off-PPI but with no within-group changes (treatment effect) (**Figure S4B**). Differences were confirmed in FD-stoppers vs. FD-starters (β= -7.22 ± 3.12, *P*= .03) and controls (β= -9.76 ± 3.01, *P*= .003) off-PPI using a cut-off of 10,000 reads.

Duodenal dysbiosis in relation to efficacy of PPI in FD patients and controls

Following the addition of the standardized (mean value= 0 and standard deviation= 1) change (Δ) in mucus-associated genera or diversity and interaction with treatment in the models of symptoms and duodenal eosinophils in FD-starters, no significant interaction effects were found for Δ*Porphyromonas*, Δ*Neisseria*, Δ*Prevotella*, Δ*Streptococcus* or ΔShannon index (**Table S6**).

Similar models were used to study associations between PPI-induced changes in host factors and microbiota in controls. The treatment*Δ*Streptococcus* interaction effect for eosinophils was explained by significantly increased eosinophils for mean -1SD (β= 1.37 ± .5, *P*= .01), mean (β= 2.39 ± .35), mean +1 (β= 3.41 ± .5) and +2SD (β= 4.43 ± .8, all *P*< .0001) changes in mucus-associated *Streptococcus* after PPI (**Table S6**). A treatment*Δ*Streptococcus* interaction effect was also found for secondary (F= 5.64, *P*= .03) but not primary bile salts (*P*= .06), with increased secondary bile salts for the mean (β= 4.24 ± 1.15, *P*= .002), mean +1 (β= 6.57 ± 1.5, *P*< .001) and +2SD (β= 8.91 ± 2.26, *P*= .001) changes in *Streptococcus* after PPI. In contrast, increased *Streptococcus* was not associated with changes in secondary (*P*= .2) or primary (*P*= .17) duodenal bile salts. Increased

Streptococcus and decreased diversity were not associated with changes in duodenal pH (*P*= .4 and *P*= .37, respectively). However, a treatment*ΔpH interaction effect was found for *Prevotella* (F= 4.27, *P*< .05) and explained by significant decreases for the mean -2 (β= -3.72 ± 1.21, *P*= .004), mean -1SD (β= -2.64 ± .77, *P*= .002) and mean (β= -1.53 ± .55, *P*= .009) changes in duodenal pH after PPI.

SUPPLEMENTARY METHODS

Sample collection

Based on pilot experiments with the BABD, the procedure was adapted before this study to prevent the evacuation of oral and gastric fluids from the working channel of the endoscope when advancing the device only in the duodenum. Thus, the sheathed and sealed (glycerol plug) BABD was already advanced when arriving at the gastric antrum, and again retracted in the working channel after the tip was visible, allowing evacuation of fluids before passage through the pylorus. After collection of the aseptic duodenal biopsy as previously described,[1] the sheath was kept in place and the forceps fully retracted, allowing introduction of the brush (diameter 1.8mm and length 230cm) through the sheath of the BABD (diameter 2.6mm and length 180cm). This technique also prevented exposure of the sheathed (but not sealed) brush device to oral and gastric fluids present in or evacuated from the working channel, in contrast to previous studies.[1,2]

Routine duodenal biopsies (D2) and fluids were processed as previously described.[3] In brief, duodenal eosinophils (H&E) and mast cells (c-kit) were counted per mm^2 in a random and blinded fashion. Transepithelial electrical resistance and paracellular passage of a fluorescein isothiocyanate-labeled 4kDa dextran (FD4, 1 mg/mL; Sigma-Aldrich, St Louis, USA) were determined in modified 3mL Ussing Chambers (Mussler Scientific Instruments, Aachen, Germany). After the endoscopy, duodenal fluids were obtained via a double-lumen naso-duodenal aspiration catheter, which was positioned in D2 under fluoroscopic control. Luminal pH was determined using a Portavo 902 PH portable pH meter (Knick, Berlin, Germany) with a BioTrode electrode (Hamilton, Bonaduz, Switzerland) before measuring primary and secondary bile salts using liquid chromatography-tandem mass spectrometry.[4] Fasting samples were used for analyses in relation to the duodenal microbiota. At baseline, *Helicobacter pylori* was excluded in gastric biopsies (Giemsa staining). The PAGI-SYM questionnaire, specific for upper GI disorders, was collected at each visit with the total score ranging from 0 (none) to 5 (very severe) over a two-week recall period.[5] Finally, a validated and online Food frequency questionnaire (FFQ) was completed to estimate the total energy and macro-nutrient (carbohydrates, fat, fiber and protein) intake at baseline and follow-up.[6]

Sample and data processing

Reaction compositions and (q)PCR settings are shown in **Table S7**. First, qPCR (Uni16S) of diluted brush (1:5) and biopsy (1:2) samples was performed using the KAPA SYBR® Fast qPCR Kit (Roche, Pleasanton, USA). Dilutions with nuclease-free water were based on pilot experiments and adapted if needed. For the microbiota analysis, the V4 region of the 16S rRNA gene was amplified with the primer pair 515F and 806R (GTGYCAGCMGCCGCGGTAA and GGACTACNVGGGTWTCTAAT, respectively),

modified to contain a barcode sequence between each primer and the Illumina adaptor sequences to produce dual-barcoded libraries.[7] Internal PCR-controls included negative (no-template) and no-primer controls to assess potential contamination of the primer plate and master mix, respectively. Positive controls included a standard diluted fecal sample on each run and predefined universal combination of bacterial strains (Zymo Research, Irvine, USA). PCR amplification was performed in triplicate and DNA concentration and fragment lengths of individually pooled amplicons were determined using a 5200 Fragment Analyzer (Agilent, Santa Clara, USA) according to the manufacturer's instructions. Following equimolar pooling and clean-up of the library with QIAquick® PCR Purification kit (Qiagen, Hilden, Germany), the final concentration was confirmed using the Qubit® dsDNA BR Assay Kit (Invitrogen) before sequencing on the Illumina MiSeq platform (500 cycles, 20% PhiX; MiSeq Reagent Kit, version 2) at the VIB Nucleomics core laboratory (KU Leuven, Belgium).[7]

Sequences were processed using the LotuS and DADA2 pipelines (v. 1.6) with taxonomic annotation formatted RDP training set 'rdp_train_set_16'.[8,9] Sequences unclassified at phylum level or annotated to the class Chloroplast or family mitochondria were removed as previously described.[10] Data from all brush or biopsy samples and negative controls (extraction blanks and no-template controls) were then filtered using the open-source R package decontam.[11] Statistical removal of contaminants was done using the prevalence-based method, which is based on the assumption of contaminants appearing in a smaller fraction of the biological samples vs. negative controls due the presence of competing true bacterial DNA. The classification threshold of 0.5 was used, allowing removal of sequences present in a higher fraction of negative controls compared to brush and biopsy samples.[11]

Statistical analysis

As there is no previous study investigating effects of PPI on the duodenal microbiome, no reasonable power analysis was possible. However, a number of 60 subjects would allow reproduction of previous findings on microbiota covariates,[12] with repeated sampling in all subjects in the current study. Between-group analysis of baseline covariates was done with Kruskal-Wallis test and post-hoc Dunn test (if applicable) for continuous data and chi-square tests for proportions. Beta-diversity and genus relative abundances were studied after CLR-transformation as required for compositional data with a minimum number of 1,000 reads and proportion of .001.[13] Univariate dbRDA was followed by a stepwise multivariate model including those variables which remained significant after adjustment (Benjamini-Hochberg FDR< .1). Permutational MANOVA (PERMANOVA) was performed with the vegan function adonis, using 10,000 permutations. Comparisons of genus relative abundance between and within groups (including spatial and temporal variation) and correlations were done for taxonomically assigned genera with a prevalence of >20%.[14] Calculation of α-diversity metrics was performed after sub-sampling to 1,000 reads using phyloseq.[15]

For linear mixed models, box-cox or logarithmic transformations of the dependent variables were done depending on normality (Kolmogorov-Smirnov test). Variables which could not be transformed, were analyzed with generalized linear

models with the identity link function. Between-group differences and the effect of initiation (controls and FD-starters) or withdrawal (FD-stoppers) of PPI-therapy were studied using planned contrasts. Following a treatment-by-group interaction effect, differences in PPI-related changes between groups were adjusted (stepdown Bonferroni). Based on our previous findings of differential effects of PPI in FD patients and controls, associations between changes in symptoms or duodenal eosinophils and the microbiota were determined in FD-starters and controls.[3] To this end, changes (Δ) in microbial variables were standardized (mean value 0 and standard deviation (SD) of 1) and entered in the model of symptoms and eosinophils, including the interaction with treatment (PPI). In case of significant interaction effects, the evolution in symptoms and eosinophils were plotted for different levels of the mean ± 1 or 2 SD changes (Δ) in microbial variables after PPI.

SUPPLEMENTARY REFERENCES

1 Shanahan ER, Zhong L, Talley NJ, et al. Characterisation of the gastrointestinal mucosa-associated microbiota: a novel technique to prevent cross-contamination during endoscopic procedures. Aliment Pharmacol Ther 2016;43:1186–96. doi:10.1111/apt.13622

2 Fukui A, Takagi T, Naito Y, et al. Higher Levels of Streptococcus in Upper Gastrointestinal Mucosa Associated with Symptoms in Patients with Functional Dyspepsia. Digestion 2020;101:38–45. doi:10.1159/000504090

3 Wauters L, Ceulemans M, Frings D, et al. Proton pump inhibitors reduce duodenal eosinophilia, mast cells and permeability in patients with functional dyspepsia. Gastroenterology 2021;160:1521-1531.e9. doi:10.1053/j.gastro.2020.12.016

4 Riethorst D, Mols R, Duchateau G, et al. Characterization of Human Duodenal Fluids in Fasted and Fed State Conditions. J Pharm Sci 2016;105:673–81. doi:10.1002/jps.24603

5 Revicki DA, Rentz AM, Tack J, et al. Responsiveness and interpretation of a symptom severity index specific to upper gastrointestinal disorders. Clin Gastroenterol Hepatol 2004;2:769–77. doi:10.1016/S1542-3565(04)00348-9

6 Matthys C, Meulemans A, Van der Schueren B. Development and validation of general FFQ for use in clinical practice. Ann Nutr Metab 2015;67:239.

7 Tito RY, Cypers H, Joossens M, et al. Brief Report: Dialister as a Microbial Marker of Disease Activity in Spondyloarthritis. Arthritis Rheumatol (Hoboken, NJ) 2017;69:114–21. doi:10.1002/art.39802

8 Hildebrand F, Tadeo R, Voigt AY, et al. LotuS: an efficient and user-friendly OTU processing pipeline. Microbiome 2014;2:30. doi:10.1186/2049-2618-2-30

9 Callahan BJ, McMurdie PJ, Rosen MJ, et al. DADA2: High-resolution sample inference from Illumina amplicon data. Nat Methods 2016;13:581–3. doi:10.1038/nmeth.3869

10 Tito RY, Chaffron S, Caenepeel C, et al. Population-level analysis of Blastocystis subtype prevalence and variation in the human gut microbiota. Gut 2019;68:1180–9. doi:10.1136/gutjnl-2018-316106

11 Davis NM, Proctor DiM, Holmes SP, et al. Simple statistical identification and removal of contaminant sequences in marker-gene and metagenomics data. Microbiome 2018;6:226. doi:10.1186/s40168-018-0605-2

12 Vandeputte D, Falony G, Vieira-Silva S, et al. Stool consistency is strongly associated with gut microbiota richness and composition, enterotypes and bacterial growth rates. Gut 2016;65:57–62. doi:10.1136/gutjnl-2015-309618

13 Gloor GB, Macklaim JM, Pawlowsky-Glahn V, et al. Microbiome Datasets Are Compositional: And This Is Not Optional. Front Microbiol 2017;8:2224. doi:10.3389/fmicb.2017.02224

14 Valles-Colomer M, Falony G, Darzi Y, et al. The neuroactive potential of the human gut microbiota in quality of life and depression. Nat Microbiol 2019;4:623–32. doi:10.1038/s41564-018-0337-x

15 McMurdie PJ, Holmes S. phyloseq: an R package for reproducible interactive analysis and graphics of microbiome census data. PLoS One 2013;8:e61217. doi:10.1371/journal.pone.0061217

16 Eisenhofer R, Minich JJ, Marotz C, et al. Contamination in Low Microbial Biomass Microbiome Studies: Issues and Recommendations. Trends Microbiol 2019;27:105–17. doi:10.1016/j.tim.2018.11.003

SUPPLEMENTARY FIGURES:

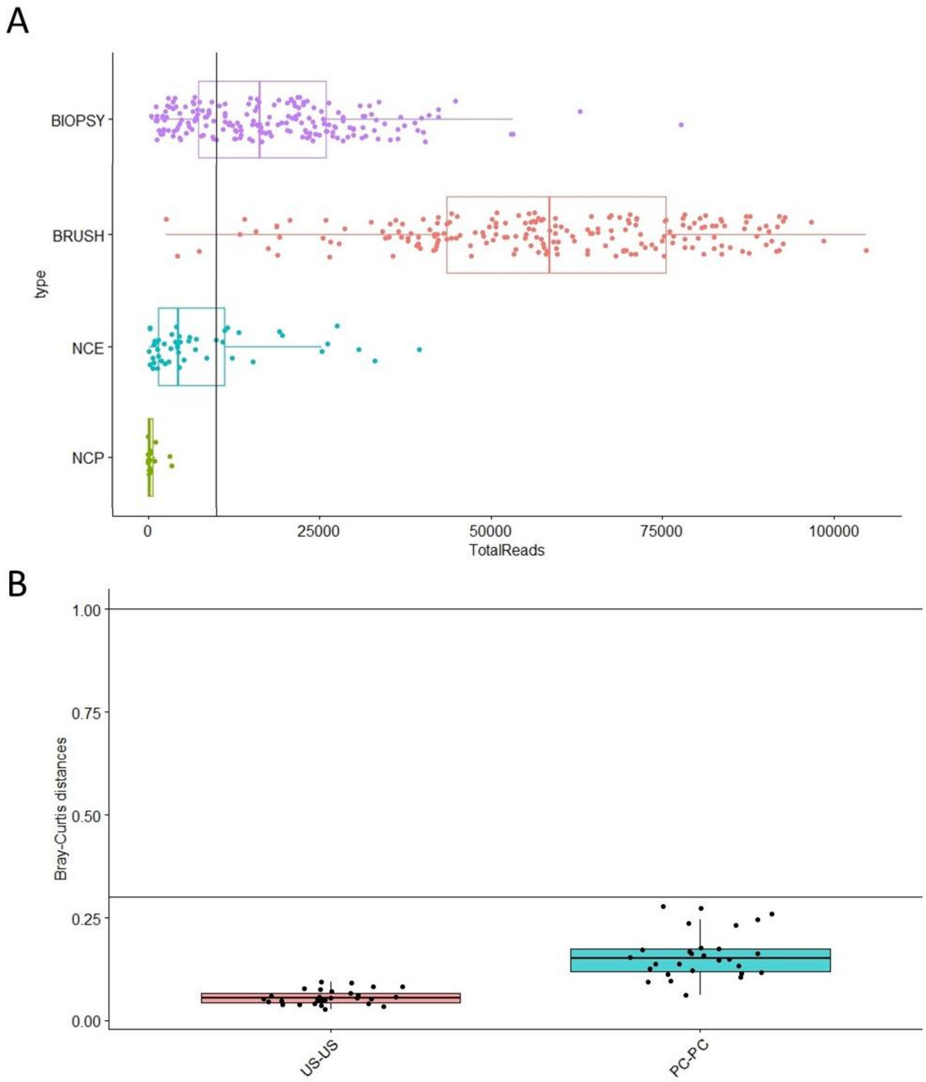

Figure S1: Quality control. A: number of reads (TotalReads) in all mucus- (brush) or epithelium-associated (biopsy) samples and negative controls are shown in relation to the additional cut-off of 10,000 reads (vertical line). **B:** Bray-Curtis dissimilarity of positive and universal controls (rarefied to 1,000 reads) are shown in relation to a maximum of 0.3 (horizontal line). NCE, negative extraction controls; NCP, negative PCR-controls; PC, positive control; US, universal standard.

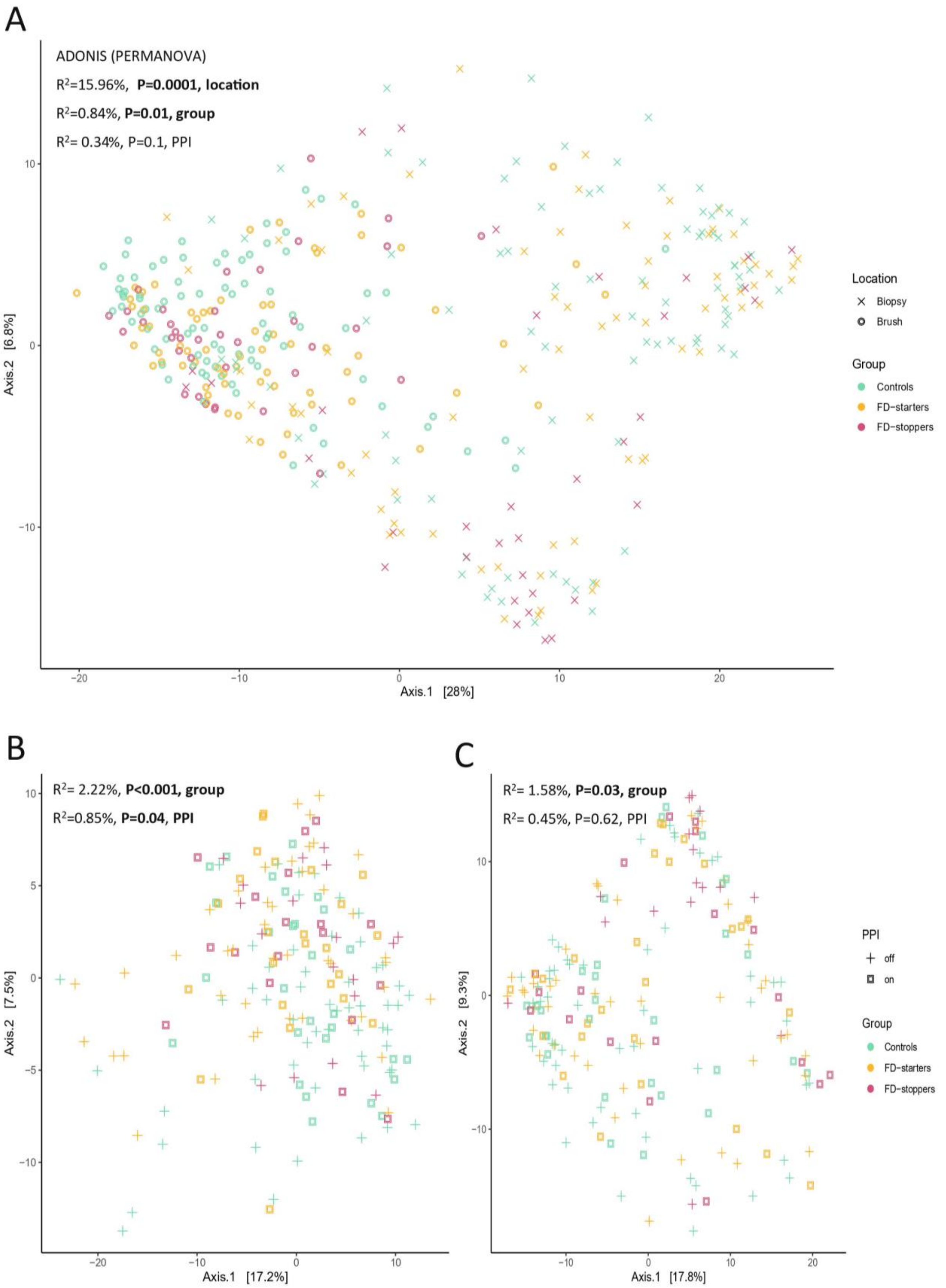

Figure S2: Duodenal microbial community variation with effect sizes of location (all samples), group and PPI-therapy. A-C Principle component analyses with the percentage of variation explained by the first 2 principal components reported on the axes for all (**A**) and duodenal brush (**B**) or biopsy (**C**) samples. Effect sizes were determined using the vegan function adonis (PERMANOVA). FD, functional dyspepsia; PPI, proton pump inhibitor.

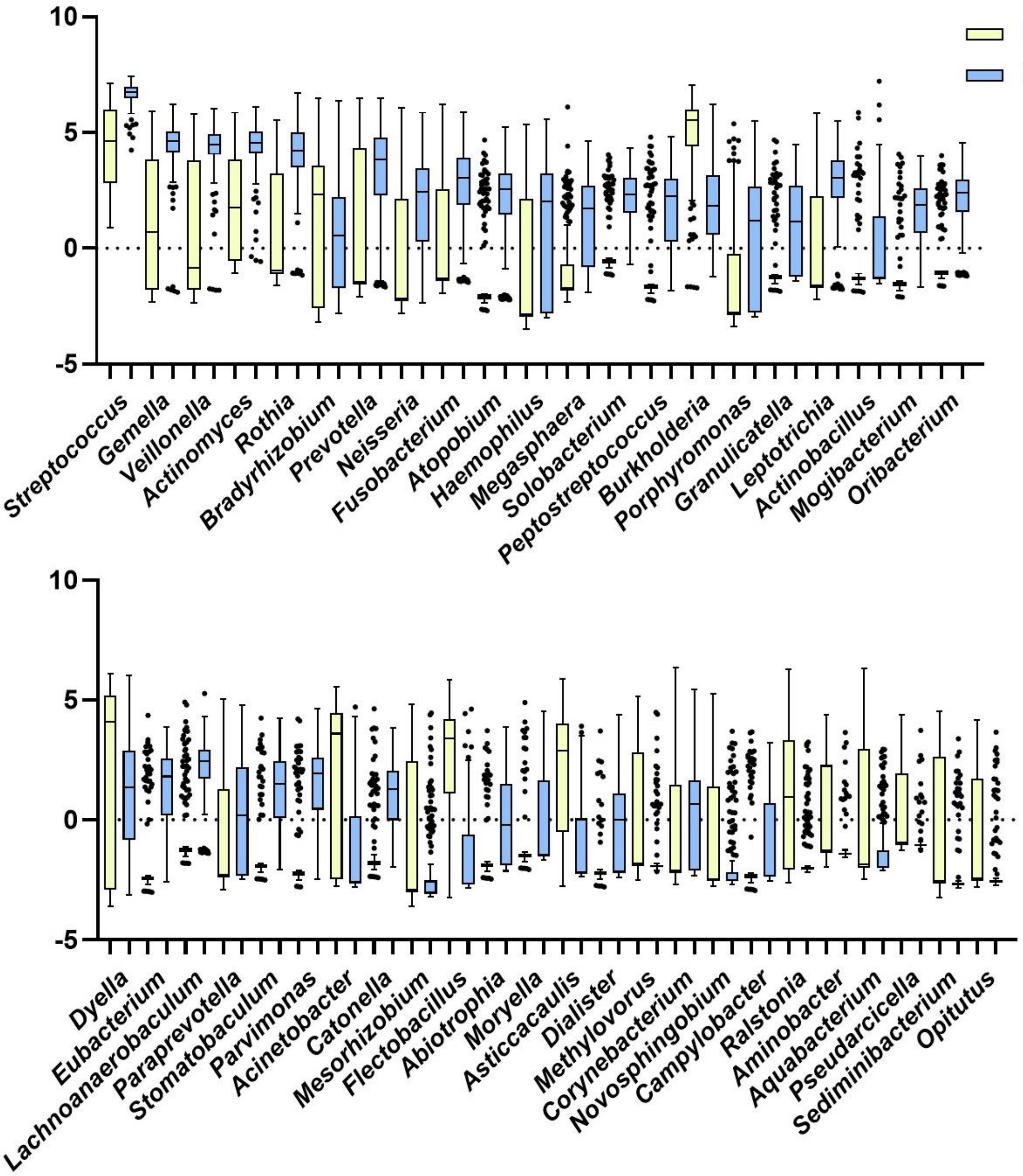

Figure S3: Spatial variation of duodenal genera. Differential genera-abundance for all paired (mucus- and epithelium-associated) samples. FDR< .1 (between-locations) for all genera (>20% prevalence).

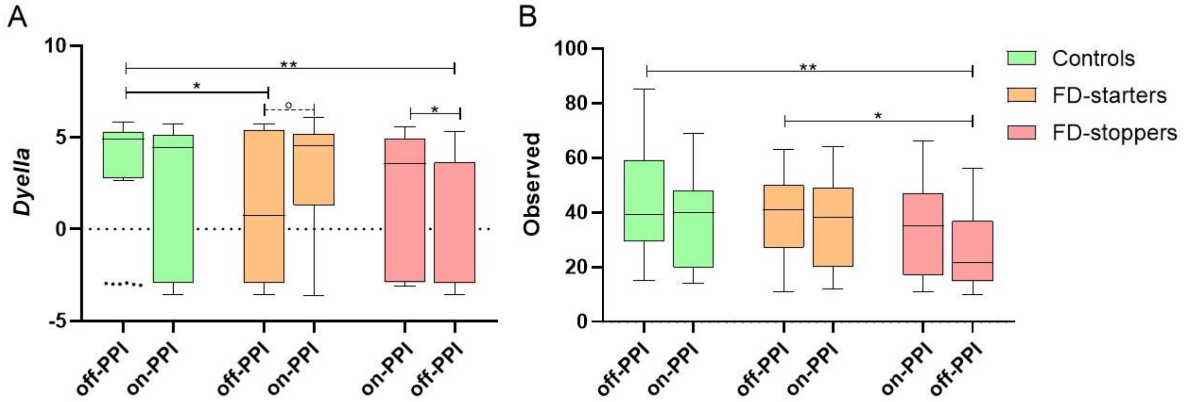

Figure S4: Relative abundances of epithelium-associated duodenal genera and diversity across groups and PPI-status. Changes in epithelium-associated *Dyella* (**A**) and Observed taxa or richness (**B**) for controls, FD-starters and –stoppers according to PPI-status. Tukey boxplots of CLR-transformed genera with median, IQR and 1.5*IQR whiskers (outliers beyond). °*P*< .1, **P*< .05, ***P*< .01. FD, functional dyspepsia; PPI, proton pump inhibitor.

SUPPLEMENTARY TABLES:

Table S1: Overview of study subjects and samples.

**Location and visit**	**Brush**				**Biopsy**			
	**baseline**	**variability**	**follow-up**	**total**	**baseline**	**variability**	**follow-up**	**total**
Subjects	77	44	75	**196**	77	44	75	**196**
Samples	75	44	72	**191**	77	44	75	**196**
Sequenced	74	43	71	**188**	76	43	74	**193**
Reads >1,000	74	43	71	**188**	76	43	73	**192**
Reads >10,000	74	42	69	**185**	51	33	46	**130**

Number of subjects and samples collected and with high-quality sequences after quality control for different minimum of reads.

Table S2: Main and interaction effects for mucus-associated genera of interest and diversity metrics.

**Effect**	**Treatment**	**Group**	**Treatment*group**
**Model information**	**F value (*P*)**	**F value (*P*)**	**F value (*P*)**
**Genera of interest:**			
Neisseria	.74 (.39)	**4.82 (.01)**	.12 (.89)
Porphyromonas	**4.45 (.04)**	**5.1 (.008)**	.89 (.41)
Prevotella	**20.33 (<.0001)**	.76 (.47)	.66 (.52)
Streptococcus	**5.44 (.02)**	1 (.37)	2 (.14)
**Diversity:**			
Observed	**6.2 (.02)**	2.7 (.07)	.29 (.75)
Chao1	.55 (.46)	.7 (.5)	.82 (.44)
Shannon	**10.89 (.002)**	.58 (.56)	1.65 (.2)
Simpson	**7.76 (.007)**	.42 (.66)	**3.3 (.04)**

Type 3 effects for linear mixed model analyses, with treatment as within- and group as between-subject factors of interest.

Table S3: Spatial variation of duodenal α-diversity metrics according to PPI-status.

Group	Controls (n= 30)		FD-starters (n= 28)		*P*-value
**Location**	**brush**	**biopsy**	**brush**	**biopsy**	
**Off-PPI**					
Observed	43.07 ± 3.33	42.03 ± 1.36	43.36 ± 7.08	36.81 ± 1.6	.66
Chao1	48.98 ± 1.91	45.43 ± 3.82	43.24 ± 2.28	47.74 ± 8.62	.59
Shannon	2.32 ± .05	2.82 ± .07 ****	2.24 ± .07	2.69 ± .12 ****	.71
Simpson	.79 ± .01	.88 ± .01 ****	.78 ± .02	.86 ± .02 ****	.72
**On-PPI**					
Observed	37.79 ± 1.9	41.43 ± 6.12	34.79 ± 1.67	42.37 ± 7.32	.71
Chao1	44.81 ± 2.71	43.58 ± 6.95	44.89 ± 2.75	45.36 ± 8.53	.99
Shannon	2.05 ± .08	2.8 ± .1 ****	1.92 ± .1	2.7 ± .12 ****	.75
Simpson	.72 ± .02	.89 ± .01 ****	.68 ± .03	.87 ± .02 ****	.96

**P*< .05, ***P*< .01, ****P*< .001, *****P*< .0001 (within-group) with lower α-diversity for mucus- (brush) vs. epithelium-associated (biopsy) samples, which was similar between groups. FD, functional dyspepsia; PPI, proton pump inhibitors.

Table S4: Temporal variation of mucus- and epithelium-associated α-diversity metrics.

Group	Controls (n= 25)		FD-starters (n= 19)		*P*-value
**Visit**	**baseline**	**variability**	**baseline**	**variability**	
**Brush**					
Observed	42.03 ± 1.36	42.24 ± 1.87	36.81 ± 1.6	38.89 ± 2.02	.45
Chao1	48.98 ± 1.91	47.93 ± 2.51	43.24 ± 2.28	43.72 ± 2.33	.68
Shannon	2.32 ± .05	2.32 ± .08	2.24 ± .07	2.27 ± .11	.82
Simpson	.79 ± .01	.78 ± .02	.78 ± .02	.78 ± .02	.72
**Biopsy**					
Observed	43.07 ± 3.33	46 ± 4.78	44.56 ± 7.25	54.83 ± 10.41	.68
Chao1	45.43 ± 3.82	49.5 ± 5.71	49.1 ± 8.83	59.36 ± 11.57	.78
Shannon	2.82 ± .07	2.85 ± .08	2.71 ± .12	2.91 ± .14	.44
Simpson	.88 ± .01	.89 ± .01	.86 ± .02	.89 ± .01	.6

No significant changes were observed for baseline vs. variability visits off-PPI (within or between groups). FD, functional dyspepsia; PPI, proton pump inhibitors.

Table S5: Duodenal α-diversity metrics before and after PPI-therapy in FD-stoppers.

Group	FD-stoppers		P_{adj}-value (controls)	P_{adj}-value (FD-starters)
**Treatment**	**On-PPI (n= 19)**	**Off-PPI (n= 18)**		
**Brush**				
Observed	37.94 ± 2.05	40.78 ± 2.72	1	1
Chao1	45.11 ± 2.64	46.89 ± 3.7	1	1
Shannon	2.12 ± .1	2.16 ± .12	.34	.28
Simpson	.75 ± .03	.73 ± .03	.07	.06
**Biopsy**				
Observed	33.21 ± 3.58	36.31 ± 11.72	.58	.58
Chao1	34.84 ± 4.06	38.71 ± 13.16	.65	.65
Shannon	2.5 ± .11	2.63 ± .18	1	1
Simpson	.84 ±.02	.87 ± .02	1	1

No significant changes were observed within FD-stoppers or between groups (P_{adj}). FD, functional dyspepsia; PPI, proton pump inhibitors.

Table S6: Interaction effects for changes in mucus-associated genera of interest and diversity.

Group	FD-starters		Controls
**Outcome**	**Symptoms**	**Eosinophils**	**Eosinophils**
**Model information**	**F value (*P*)**	**F value (*P*)**	**F value (*P*)**
**Mucus-associated (brush)**			
• Δ*Porphyromonas*	.12 (.74)	.84 (.37)	2.5 (.13)
• Δ*Neisseria*	1.6 (.22)	.06 (.81)	.62 (.44)
• Δ*Prevotella*	0 (.96)	.07 (.79)	.16 (.69)
• Δ*Streptococcus*	.32 (.58)	2.37 (.14)	**8.18 (.008)**
• ΔShannon	.64 (.43)	1.45 (.24)	1.65 (.21)

Linear mixed model analyses including the standardized PPI-induced change in mucus-associated genera of interest or diversity in the model with symptoms (FD-starters) and duodenal eosinophils (FD-starters and controls). FD, functional dyspepsia; PPI, proton pump inhibitors.

Table S7: Reaction compositions and (q)PCR incubation settings.

Method	Component	Volume (µl)	Total (µl)
qPCR (KAPA SYBR)	Master Mix (2X)	10	20
	ROX Low (50X)	0.4	
	Forward primer (10 µM)	0.4	
	Reverse primer (10 µM)	0.4	
	Nuclease-free water	6.8	
	cDNA template	2	
PCR (AccuPrime)	PCR Buffer II (10X)	3	30
	Taq DNA Polymerase High Fidelity	0.1	
	Primer mix (10 µM)	2	
	Nuclease-free water	22.9	
	cDNA template	2	

Method	Description	Temperature (°C)	Time (min:s)	Cycles
qPCR (KAPA SYBR)	**Enzyme activation**	95	0:20	/
	**Denaturation**	95	0:03	45
	**Annealing**	60	0:30	
PCR (AccuPrime)	**Initial denaturation**	95	3:00	/
	**Denaturation**	95	0:45	33
	**Annealing**	60	1:30	
	**Elongation**	72	1:30	
	**Final elongation**	72	5:00	/
